# Community education program on autism spectrum disorder: a case study of an ecosystem intervention involving family, school, NGO, and community stakeholders

**DOI:** 10.3389/fpubh.2026.1789943

**Published:** 2026-04-21

**Authors:** Cecilia Hok Man Wong, Camille Kuen Yu Chan, Paul Wai Ching Wong

**Affiliations:** 1 Faculty of Social Sciences, The University of Hong Kong, Hong Kong, Hong Kong SAR, China; 2 Department of Social Work and Social Administration, Faulty of Social Sciences, The University of Hong Kong, Hong Kong, Hong Kong SAR, China

**Keywords:** autism spectrum disorder (ASD), community education and awareness, cross-sectoral approach, ecological analysis, neurodevelopmental disorders, neurodiversity, public health promotion, stakeholder engagement

## Abstract

**Background:**

This paper documents the JC A-Connect: Jockey Club Autism Support Network community education program, which was a decade-long, territory-wide, and cross-sectoral initiative in Hong Kong, China. It was created in response to the rise of students with autism spectrum disorder (ASD) in mainstream schools which posed challenges to support their adaptation. The program came timely as the number of autistic students doubled during the project implementation period.

**Program:**

Unlike other initiatives that directly served the autistic individuals, this program delivered at the wider social networks aiming to promote social inclusiveness and empower the family, school, and community stakeholders. Three core strategies were formulated: (1) Iconic Character and Storybooks—An iconic character, Bling Bling, was created by the company of Mr. Men Little Miss, and five storybooks were written to equip readers with strategies to communicate with autistic children. (2) Media Promotion and Online Learning—Traditional media and social media were employed to disseminate the sharing from experts and families with autistic children to offer a balanced and constructive understanding of ASD. Free online learning resources were created. (3) Events for Public Engagement and Community Inclusion—Events were organized to educate the public, empower professionals, create positive community experiences for families with developmental delays and disabilities, and encourage mutual appreciation and respect among students.

**Outcomes and reflections:**

The program organized 56 public events, and created 2 online courses, 47 media promotion, 12 newsletters and over 60 free resources. Participants reported that the program helped them learn about ASD, and they became supportive toward building an autism-friendly environment. There were four factors leading to positive outcomes: The use of an ecological model for program planning and implementation, a strategic stakeholder management plan, an attractive icon, and a sustainability mindset.

**Conclusion:**

Through this case study, we seek to share our experiences in developing a cross-sectoral health program and to inspire health professionals and policy makers to consider intervening at social systems for systemic changes in communities. We acknowledge that the development, implementation, and sustainability of our strategies require multidisciplinary expertise and financial investment, and this calls for strategic planning, community-wide participation, and shared ownership.

## Introduction

Autism is a neurodevelopmental challenge of global concern. Estimated by Zeidan et al. (1) in their systematic review, the median global prevalence has reached 100 per 10,000 individuals. Talantseva et al. (2) meanwhile conducted a stratified meta-analysis. They reported that the global prevalence rates of autism spectrum disorder (ASD), autistic disorder, Asperger syndrome, and atypical autism and pervasive development disorder not otherwise specified stood at 0.72%, 0.25%, 0.13%, and 0.18% respectively. Sharing across these reviews are two alarming messages: (1) For every 140 persons, there is at least one autism case, and (2) the number of confirmed cases keeps rising. In response to this growing prevalence, the United Nations and the World Health Organization endeavor to integrate autism into the broader public health policies and disability legislation. Through initiatives such as the World Health Assembly WHA Resolution 67.8, the Convention on the Rights of Persons with Disabilities, and Autism Awareness Day, they seek to strengthen national healthcare capacities and ensure socioeconomic inclusion for neurodivergent populations through systemic advocacy and evidence-based interventions (3–6).

From the literature, Hume et al. have identified 28 evidence-based interventions for children, youth and young adults with autism, they together address challenges in domains ranging from cognition, social communication, motor skills to vocational development (7). Most of these are direct interventions for autistic individuals aiming to promote their functioning and reduce atypical behaviors. While their effectiveness in creating positive changes has been confirmed through research (8–10), we advocate that programs that target the wider social network for social inclusiveness are equally important. This is especially true as autism is characterized by difficulties in social communication that involves people in the social circles; by cultivating an autism-friendly culture, we can indirectly support the social functioning and wellbeing of autistic individuals (11, 12).

Building an inclusive culture is one of the proposals put forth by the neurodiversity advocates (13, 14). In recent decades, the neurodiversity movement has emerged as a strong force in advocating for the welfare of people with atypical development (15). It urges the communities to use a social model to comprehend the experiences of people with autism, intellectual disabilities or mental health challenges, and examine how the environment creates and sustains disabilities (15–17). In addressing the values of neurodiversity for clinicians and medical researchers, Sonuga-Barke and Thapar suggest that this paradigm shifts our attention from the biopsychological basis of disorders to the limitations posed on autistic individuals by the physical and social settings, which undermine their self-esteem and mental health while creating stigmas (18). To date, stigmas toward autism still prevail across arenas, and the lack of inclusiveness calls for systemic changes in the society (19–21). Research shows that autistic children generally have a lower participation in school activities than their peers with or without disabilities, and the parents long for more peer interactions for their children in schools and in communities (22). With an empowered social system that embraces inclusivity, we are hopeful to witness more flourishing relationships with and among autistic individuals.

Indeed, public health professionals have long been called to employ an ecosystem approach to health, which is also known as “ecohealth” (23). It situates wellbeing in the context of social-ecological dynamics and directs our attention to the influences of social systems (23, 24). For example, adopting a socio-ecological lens, Brisendine et al. (25) discovered that the insurance coverage and economic hardship of a family would affect the caregivers' decisions of whether to report their children's autism diagnosis. Taking the family system into perspective, Wong et al. (26) found that family dynamics could augment or negate the effects of parent-implemented early intervention. Using an ecological approach to identify and modify social determinants offers health professionals new insights for interventions.

In this paper, we document a public health intervention in Hong Kong, China that employed the ecological system theory for needs analysis and strategy development. As a territory-wide community education program, this intervention aimed at supporting autistic children and youth by equipping their communities with the knowledge about ASD and an inclusive attitude toward autistic individuals, their families, and their paid and non-paid caregivers. Swiezy et al. (27) shared a similar idea of intervening at the social system, and they suggested offering specialized training to the care providers across home, medical and educational settings. The intervention described in this article transcended their proposed scope by including not only the care providers but also the peer students in schools and the general public.

## Context

In view of the rise of autistic students in mainstream public schools in Hong Kong under the “Whole School Approach,” which posed challenges to schools and families to support their adaptation in the school setting, the Hong Kong Jockey Club Charities Trust (HKJC) initiated and funded a project called “JC A-Connect: Jockey Club Autism Support Network” (JC A-Connect) in 2015 (28). With HKJC spearheading the development and connecting organizational stakeholders across sectors, three working teams were set up.

The School Support Team was led by educational psychologists in the Department of Psychology of The University of Hong Kong (HKU). They developed training and learning materials for primary and secondary school teachers and empowered local NGOs to provide trainings for teachers. Their service model had been implemented in over 500 schools and was later adopted by the Education Bureau from the school year of 2021/22 (28).

The Family Support Team was led by clinical psychologists in the Department of Social Work and Social Administration, HKU. They provided direct trainings to caregivers of children with ASD, mobilized local NGOs to provide early intervention services for families, and introduced the World Health Organization's Caregivers Skills Training for families of children with developmental delays or disabilities to Hong Kong (26, 29, 30).

The Community Education Team was managed by a program coordinator in the Faculty of Social Sciences, HKU. They worked with the other two working teams and some local NGOs to co-design and implement the community education program studied in this paper.

This program was a decade-long, territory-wide and cross-sectoral initiative with its scope and complexity transcending the earlier projects in Hong Kong. Throughout the project period from 2015 to 2025, we witnessed a surge of autism cases, especially among children, in Hong Kong. As revealed in the general household survey conducted by the Census and Statistics Department, the overall prevalence rate of ASD increased from 0.1% in 2013 to 0.3% in 2020; for children aged 14 or below, 14 out of 1,000 were diagnosed with ASD in 2020 (31). A recent study published in 2025 reported that the estimated prevalence even rose to 2.57% among children and youth aged 6–17 (32). In the academic year of 2012/13, there were only 4,150 autistic students studying in the mainstream primary and secondary schools, this figure climbed to over 10,000 in 2023 (33, 34). The sharp increase in ASD cases was influenced by various factors such as an increase in awareness of autism in the communities, the broadening of diagnostic criteria, the strengthening of training for local healthcare professionals, the advancement in diagnostic methods, and the government's commitment in reducing the waiting time for assessments (35–39). Although these changes boosted up the number of confirmed cases as they brought more autism cases to light, the collective efforts have made early diagnosis and intervention possible. The community education program together with other services offered by JC A-Connect came timely to meet the growing demand.

## The JC A-Connect community education program

The JC A-Connect community education program has two overarching goals: (1) to raise public awareness of ASD, promote inclusivity and create an autism-friendly culture; and (2) to empower the stakeholders in the social circles of autistic children with knowledge about ASD and skills to promote the functioning, development and wellbeing of autistic children.

To design a multi-level community intervention, an ecological needs analysis was conducted to identify the challenges faced by autistic students and their families at the inception of JC A-Connect.

In schools, these young people often encountered difficulties in studies and in interpersonal relationships (40). Being weaker in Theory of Mind—the ability to make sense of one's own mental states and others' (41)—they struggled to apprehend the social messages and predict what could have happened before an incident, this posed challenges for them to follow instructions from teachers and to make friends with peers. Some even reported being bullied by their classmates, some were misunderstood by their teachers and social workers as they failed to explain their actions. At the same time, the frontline school staff had their own struggles. Those with less knowledge about ASD and less experiences working with autistic students found it more challenging to make sense of the atypical behaviors and the limitations arising from the deficits, hence they were more likely to punish these students (42). However, with more knowledge and experiences, they could adjust their expectations and develop more coping strategies.

**Figure 1:**
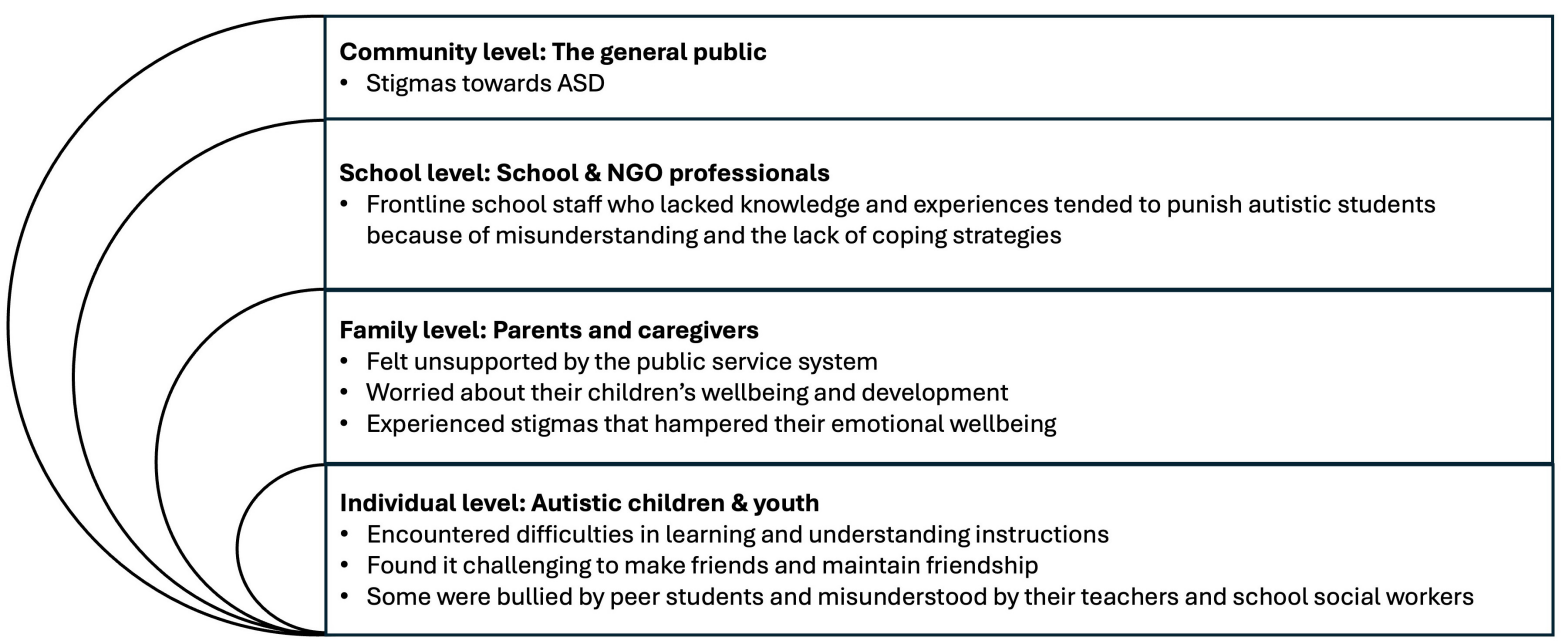
An ecological analysis of the needs of autistic children and their families in Hong Kong at the inception of JC A-Connect.

At home, the parents felt unsupported by the public system (43). They found it difficult to access timely and useful information about ASD, the waiting time for the initial assessment was long, and there was a lack of post-diagnosis service which left them feeling helpless and stressful. They also worried about their children's academic performance, school experiences and career prospects (44, 45). Meanwhile, they were troubled by the blames from themselves, their families and the communities, all these stigmas hampered their emotional wellbeing (43–46). Under tremendous pressure, some resort to abandon, neglect or hide their children from people with prejudice (42). Figure 1 summaries the challenges faced by the autistic students and their families in an ecological model.

Addressing the identified needs, three core strategies were devised: (1) Iconic Character and Storybooks, (2) Media Promotion and Online Learning, and (3) Events for Public Engagement and Community Inclusion. They targeted at the general public, school and NGO professionals, students and families.

### Strategy 1: iconic character and storybooks

**Figure 2:**
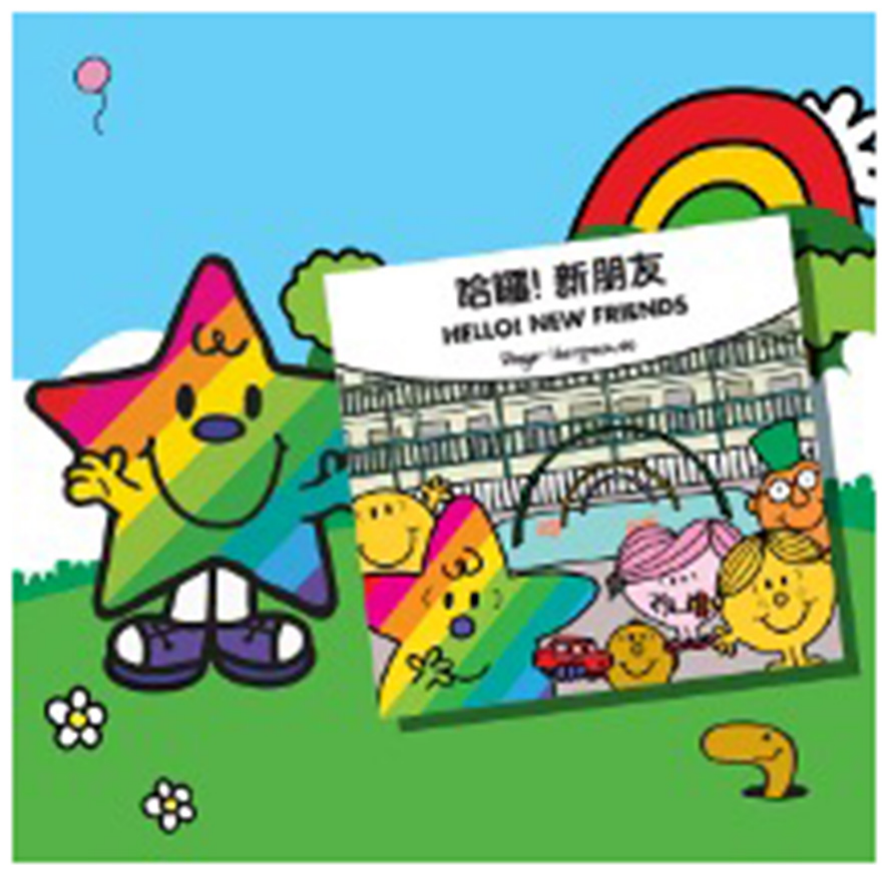
Bling Bling character and the book design of “Hello! New Friends.”

As an initiative involving a wide range of organizational partners, it was a challenge to align program image across organizations and events. A few years into its operation, the program leaders realized that creating a common icon would resolve this problem. Through HKJC's connections, the Community Education Team partnered with The Hargreaves Organization International Productions (THOIP)—the company of Mr. Men Little Miss—to create a new character “Bling Bling” in 2021. Ambassador Bling Bling was presented as a boy with ASD who had a star-shaped and rainbow-colored body (Figure 2).

For an effective icon for health campaigns, research reveals that it has to be *interesting, simple, familiar, recognizable, concrete*, and a *close* representation of the contents it communicates (47); these were the characteristics Bling Bling possessed. First, it shared the *interesting* drawing style of Mr. Men Little Miss. Second, it was drawn with *simple* lines upholding the spirit of minimalism. Third, it shared the features of Mr. Men Little Miss characters which most people found *familiar*. Fourth, the multi-colored and star-shaped design was distinctive and *recognizable*. Fifth, the character contained the features of a face, hands, legs and shoes which *concretely* portrayed a child figure. Sixth, the rainbow colors had a symbolic meaning of the autism spectrum which was a *close* representation of ASD.

While other health initiatives mainly utilized icons to share simple and direct health information, this program used Bling Bling as the main character to develop stories. Five storybooks were published: *Same among the Differences* (2021), *Go to School* (2022), *Hello! New Friends* (2022), *Bling Bling's birthday* (2023), and *Bling Bling's Adventure* (2023). They were written by the School Support Team, and each described a common atypical behavior of autistic children. Through explaining the motives of Bling Bling and illustrating how he and his friends cultivated mutual understanding, these stories helped readers decipher the messages behind the atypical behaviors and learn how to communicate with autistic children effectively. As emphasized by the main author, Dr. Kathy Wong, an educational psychologist specializing in ASD, “We don't want to amplify the symptoms. We want to focus on the reasons and needs behind the behaviors.” Figure 2 shows a book cover.

These storybooks, which were filled with interesting and educational contents, were freely distributed to the public, schools, NGOs, and families through events and organizational partners. The books served as an important tool to support non-specialists, families and students to learn about the needs of autistic children thus promoting understanding, acceptance and social inclusiveness. Meanwhile, they also supported the autistic children to learn about social norms and the ways to listen to others and express themselves.

### Strategy 2: media promotion and online learning

The second strategy was to create multimedia learning resources tailored for the public, professionals and families.

For the public, the three working teams and some local NGOs mobilized their clinical expertise and interviewed some families with autistic children to create educational materials. These materials were then distributed through traditional media and social media such as newspapers, TV, radio, YouTube, Instagram, and project newsletters. These media promotions served to counteract the misrepresentation of ASD and to eliminate the general biases and stigmatization in the existing media (48). Also, by portraying the positive aspects of ASD alongside the challenges faced by autistic children and their caregivers, this program portrayed a balanced and constructive understanding of autism.

For school and NGO professionals such as teachers, social workers, counselors, occupational therapists and speech therapists, the School Support Team developed an interactive eLearning course that could be accessed freely at the project website. This evidence-informed course adopted scenarios in the local school setting to educate professionals about the characteristics of ASD and equip them with effective emotional management and social skills training strategies. It particularly supported professionals who had not yet received specialized training on ASD. Some local NGOs also developed other specialized resource packages such as teaching manual for child-care workers, handbook of helping autistic children in mainstream schools, and activity manual for peer support for autistic children.

For families, the School Support Team developed another free eLearning course for parents with autistic children studying in primary and secondary schools. This course was similar to the one for professionals, but it had been adapted to the family setting to meet the needs of caregivers. The Family Support Team and some local NGOs also developed other learning packages for families with children from nursery to secondary school. These resources together addressed concerns ranging from parent-child relationships, home-based training, emotional management to academic skills training, and were available at the project website.

Since holding an online platform involves web hosting and administrative costs, the Faculty of Social Sciences, HKU volunteered to take over the contents and host the project website (https://www.socsc.hku.hk/JCA-Connect/en/) to sustain the efforts beyond the funding period.

### Strategy 3: events for public engagement and community inclusion

In addition to creating educational materials, this community education program also organized five types of events: Events for public engagement, public involvement, community experiences, capacity building, and peer relationships.

The events for public engagement aimed at attracting the public's attention to the JC A-Connect project thus arousing the public awareness of ASD and promoting social inclusiveness. Examples of these activities were “JC A-Connect x Star Ferry: Curiosity Ferry Ride” which had the images of Bling Bling painted on a ferry, “Bling Bling Tram Tour” with Bling Bling painted on trams, and “Bling Bling x Mr. Men Little Miss Exhibition” at the Hong Kong Book Fair for sharing information about ASD and distributing storybooks.

The events for public involvement were activities that community members could participate in to learn about ASD while having fun with families with autistic children. Examples of these activities were “Ocean Park x Mr. Men Little Miss: Unique Summer Party” with activities for families at a theme park, “JC A-Connect Family Fun Day” with indoor and outdoor performances and activities, and “Graffiti at Kam Tin Mural Village.”

The events for community experiences were activities organized exclusively for families with children with developmental delays or disabilities, examples included the “JC A-Connect Art Jamming” and “Pastel Nagomi Art Parent-Child Workshop.” Being aware that many of these caregivers hesitated to bring their children to public events, these activities were designed to offer the families a safe environment to experience community activities. For instance, in the “JC A-Connect Movie Day” events, children and caregivers were allowed to speak and walk in the theater while the movies were showing.

The events for capacity building aimed at offering useful information for the school professionals, NGO professionals, and caregivers so that they could provide timely support for autistic children. These activities include conferences, seminars and webinars.

The events for peer relationship were drama performances at local kindergartens and primary schools. These dramas were developed based on the Bling Bling storybooks and delivered through clown art. Throughout the performances, the actors interacted with the students to help them learn about ASD and develop mutual respect and care. These events served to cultivate an inclusive culture in the school environment.

The public engagement and community inclusion events were mainly coordinated by the Community Education Team with the support from other working teams, NGOs, business partners and school partners. The activities functioned as a means to engage a wide spectrum of organizational and individual stakeholders through partnership and participation thus creating synergy.

### Overview of the strategies: three levels of engagement

**Figure 3:**
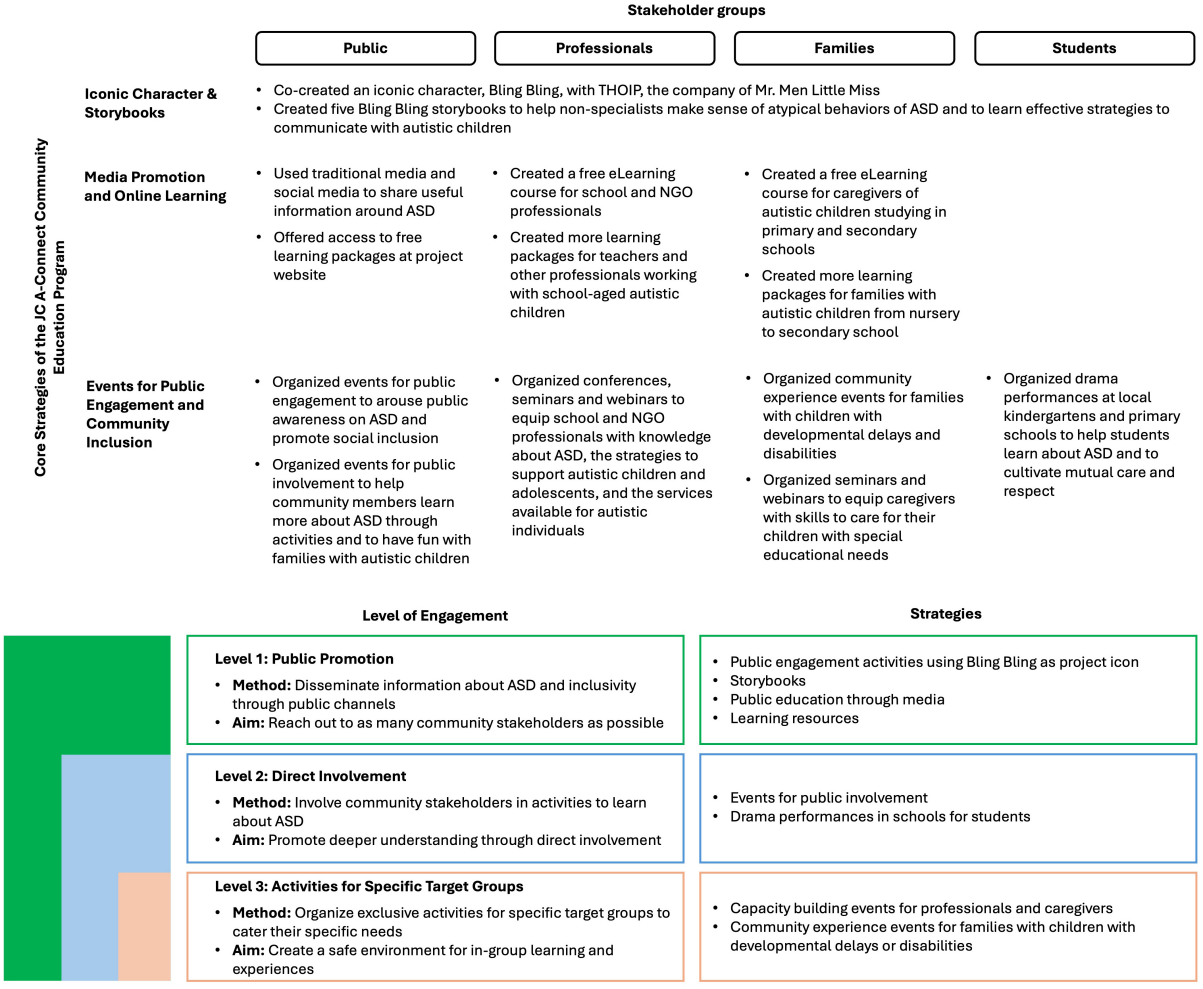
The core strategies of JC A-Connect community education program.

The strategies of JC A-Connect community education program sought to catalyze changes in communities through three levels of engagement (the higher the level, the higher the engagement). *Level 1: Public Promotion* was about disseminating information through public channels with an aim to reach out to as many community members as possible. *Level 2: Direct Involvement* was about providing community stakeholders the opportunities to learn about ASD and inclusiveness through participating in activities. *Level 3: Activities for Specific Target Groups* were events organized exclusively for professionals, students or families with children with developmental delays or disabilities so that they could have a safe environment for learning and community experiences. Figure 3 summarizes the strategies.

## Outcomes

Over the years, the JC A-Connect community education program had achieved the followings:

- Organized 42 public events with over 63,000 attendees.
- Organized 14 professional development events with over 3,900 beneficiaries.
- Created 2 online learning courses for parents, professionals and the general public with over 1,400,000 view times.
- Produced 15 TV/radio interview programs and 32 newspaper articles with over 28,000,000 viewers.
- Produced 12 newsletters reaching out to 592,000 readers.
- Created a project website which allowed the public to access over 60 free resources including learning packages, animation videos, and video recordings of past training.

Table 1 presents the accomplishments of the JC A-Connect community education program alongside the summaries of strengths and considerations. From the community events, 701 surveys were completed by the family participants. Ninety-six percent of them agreed that the events helped them know more about the characteristics of ASD, and they became more supportive toward social inclusion. Feedback form was also collected at the professional development events. While different conferences, seminars and webinars had different focuses, a majority of the participants agreed that these events helped them understand more about the latest development of services for autistic students, the strategies to support the learning and social development of autistic children and adolescents, and the practice of social inclusion in school and in community settings.

**Table 1:** Accomplishments of JC A-Connect community education program.

Core strategies	Accomplishments	Strengths and considerations
Iconic character and storybooks	•Bling Bling character •5 Bling Bling storybooks	**Strengths:** •Support non-specialists, families and students to learn about the characteristics of ASD and effective strategies to communicate with autistic children •Support autistic children to learn about social norm and the ways to listen to others and express themselves •Promote understanding, acceptance, respect and social inclusiveness **Considerations:** •Costs involved in character design, storybook design and printing •Copyrights issues
Media promotion and online learning	•2 online learning courses for professionals and caregivers with over 1,400,000 view times •15 TV/radio interview programs and 32 newspaper articles with over 28,000,000 viewers •12 newsletters reaching out to 592,000 readers •A project website which allowed the public to access over 60 free resources including publication of learning packages, animation videos introducing ASD, and videos of past training	**Strengths:** •Make useful information freely available •Foster a positive, inclusive and supportive culture •Use reachability and web contents usage to estimate the influences **Considerations:** •Require continuous and systematic quantitative and qualitative evaluation to gauge the effectiveness and impacts •Costs involved in resources development, marketing, media promotion and web hosting
Events for public engagement and community inclusion	•42 public events with over 63,000 attendees •14 professional development events with over 3,900 beneficiaries	**Strengths:** •Engage a wide spectrum of organizational and individual stakeholders through participation and partnership thus creating synergy. •Offer tailored learning opportunities to meet the needs of specific target groups •Offer fun and attractive activities to create a welcoming learning environment •Create safe environment for target groups to learn and to experience social inclusiveness **Considerations:** •One-off events •Costs involved in event organizations

The active participation of the stakeholders and the self-report surveys revealed that the program had met its objectives by successfully arousing social awareness, promoting social inclusiveness, and equipping families and professionals with knowledge and skills to promote the development of autistic children.

This program was developed based on community engagement principles. To date, no academic research has been conducted to systematically evaluate it and gauge its impacts on students, families, schools, NGOs, and communities; therefore, the main purpose of this case study was not to make a claim of the program effectiveness but to share the our lessons learnt.

## Discussion

Reflecting on over 10 years of program evolvement, we observed that there were four significant factors supporting its development and scaling up.

### Factor 1: the use of an ecological model for program planning and implementation

With a heart to provide holistic support to autistic children and their families, this program set out to identify their needs across social settings. The use of an ecological lens for planning enabled the program leaders to map out the stakeholders and intervention opportunities in different arenas, and this formed a solid foundation for building an interdisciplinary and cross-sectoral service model.

On interdisciplinary collaboration, the program leaders were aware that it required multidisciplinary expertise to meet the needs of stakeholders across home, school and community settings, hence they mobilized educational psychologists, clinical psychologists, and social workers to develop the interventions. On cross-sectoral collaboration, the program leaders preceived it important to encourage participation from different parts of the society to create synergy and shared ownership for cultural changes. Therefore, they developed partnerships spanning across academia, schools, NGOs and local businesses.

As a project of unprecedented scale and scope, it was a constant challenge to clarify the roles of different organizational partners and to coordinate among organizational and individual stakeholders. It took time and ongoing exploration for the project teams to devise a clearer picture for project development and strategy implementation. In this evolvement process, the ecological model was of help to identify the concerns, needs and strengths of different parties.

### Factor 2: the strategic stakeholder management plan

**Figure 4:**
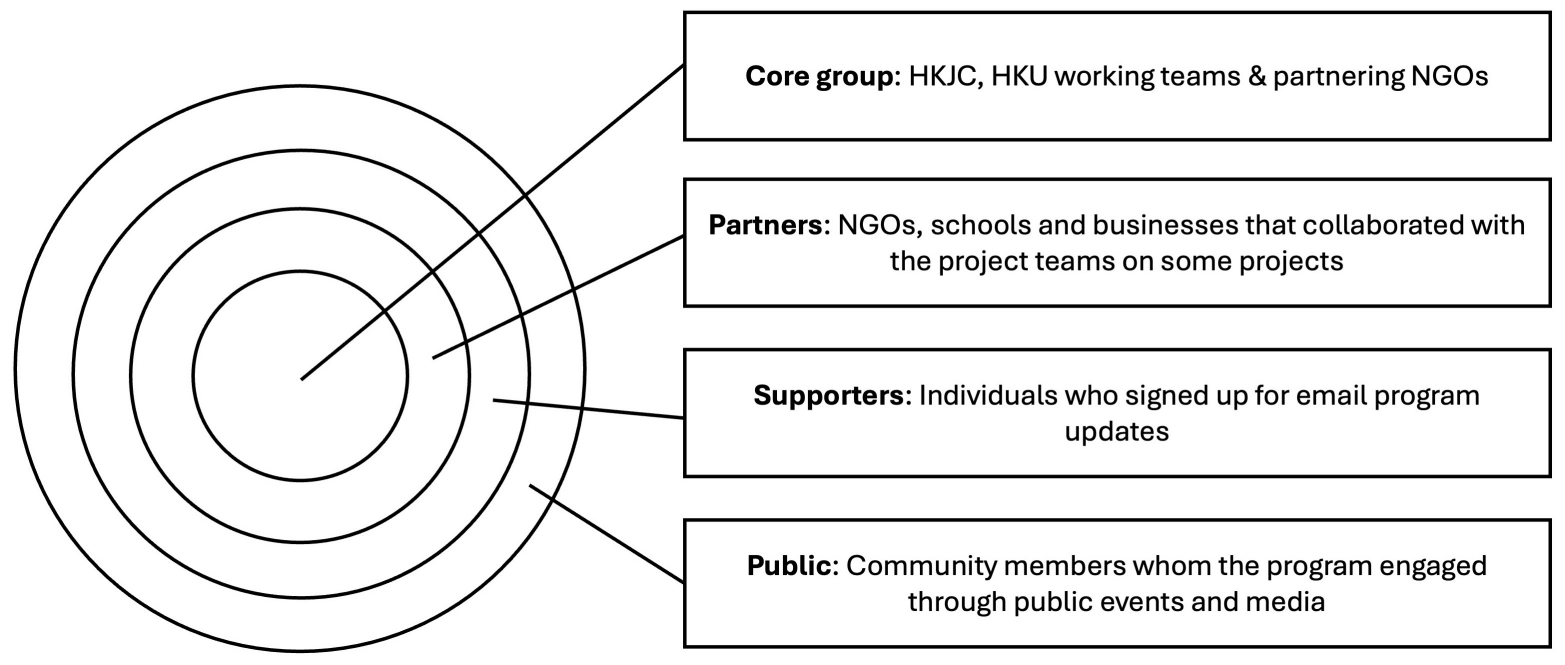
JC A-Connect community education program's strategic stakeholder management plan.

As this program involved a huge amount of organizations and individual stakeholders, it was of importance to have a strategic plan for effective engagement and management. This program stratified the stakeholders into four levels (Figure 4).

At the center was the core group which encompassed the HKJC, HKU working teams and the partnering NGOs. This group was the key driver of program development who was responsible for planning the program development and scaling up, recruiting partners, managing and monitoring the program implementation. The second inner layer included the partners who collaborated with the program teams on some projects. They were the NGOs and schools that sent their staff for capacity building, the schools participated in the drama performance events, and the organizations and businesses which supported the public events. This group directly participated in the program activities, had more exposure to the inclusiveness concept, and had the potentials for further collaboration. The third group was the subscribers who signed up for program updates. These individuals were the staff of the partnering organizations, event participants, and community members who were interested in the program. They were the supporters of the program and had the potenials to serve as ambassadors of the program in their social circles. The outer group was the general public whom the program engaged through public events and media promotion.

With this stratification, the program leaders could formulate strategies to build different groups of organizational and individual ambassadors and to allocate resources accordingly.

### Factor 3: the use of an attractive icon

Introducing Bling Bling character as the program icon had proved to be a strategic move. It helped capture the attention of the event participants and aroused curiosity for the program. In the public events, people were excited to take photos with the Bling Bling picture boards and to receive Bling Bling souvenirs, and they tended to stay longer at the exhibition and promotion booths to learn about ASD and social inclusion. This icon was also useful in standardizing the program's image across organizations, and this strengthened the program's market positioning. More, using Bling Bling stories and dramas to portray the experiences of autistic children helped make the messages vivid and easy to understand and memorize.

### Factor 4: having sustainability as an end goal in program planning

From the beginning, the program leaders were aware that the funding would one day be exhausted, hence they had sustainability as a key consideration in their planning. To sustain the impacts of the program, the leaders opted to invest heavily in training school and NGO professionals and in developing learning packages so that the professionals could continue to mobilize their learning to serve their communities and make use of the resources for service delivery after the funding period. Through these educational initiatives, the program aimed at building the professional capacities that would serve the community in the long run.

### Limitations and possible development

While the JC A-Connect community education program had launched different endeavors to serve different target groups, it still fell short to meet all the needs of the program stakeholders. This is especially true when ASD contains a wide spectrum of disabilities, and the learning materials had limited power to address all the challenges in different scenarios. This limitation was reflected by the continuous requests from professionals and families for more training by the program leaders and operators. Another limitation was a lack of involvement of autistic individuals in program planning and implementation. This could leave important concerns and needs unaddressed especially if we want to minimize or remove the constraints in our social settings, protect the welfare of autistic individuals and integrate autism into the broader public health policies and disability legislation.

As suggested by Smith et al., a community case study has the potentials to support the development of evidence-based practice (49). Based on our experiences, we propose four program and research directions. First, systematic reviews and qualitative research are needed to explore the possible theories of change for program design. This case study has offered valuable insights into what could be possible and useful for different client groups, the future initiatives can benefit from examining if these strategies are useful in their contexts. Second, evaluation indicators that can address both the program-level and activity-level objectives are yet to be developed to facilitate high-level program evaluation while monitoring the quality of activities. Third, a systematic program evaluation to cross-examine the theories of change in the actual implementation at different levels will inform the development and refinement of interventions and policies. Fourth, more involvement of autistic individuals in the design, implementation and evaluation of program is needed to ensure program's comprehensiveness and fairness. This also promotes social inclusiveness and supports the advocacy of neurodiversity paradigm of ASD.

## Conclusion

This case study documents the strategies of and learnings from a cross-sectoral, territory-wide and decade-long community education initiative on ASD in Hong Kong, and discusses the use of a socioecological model, a multi-leveled stakeholder management plan, a centralized program icon and sustainability mindset for supporting program development. Through sharing our experiences in developing a multi-level health program, we seek to inspire health professionals, community professionals and policy makers to consider intervening at the social systems for systemic changes in communities.

We acknowledge that the development, implementation and sustainability of our strategies require multidisciplinary expertise and financial investment, and this calls for strategic planning, community-wide participation, and shared ownership.

## Data Availability

The datasets presented in this study can be found in online repositories. The names of the repository/repositories and accession number(s) can be found below: the information of this community education program can be accessed at https://www.socsc.hku.hk/JCA-Connect/en/.
